# 5th International Multithematic Scientific Bio-Medical Congress (IMBMC), Nicosia, Cyprus, 2–4 November 2017

**DOI:** 10.1038/s41419-018-0393-4

**Published:** 2018-03-02

**Authors:** Anthony Lisacek-Kiosoglous, Andreas Georgiou, Ioannis Patrikios

**Affiliations:** School of Medicine, European University Cyprus, Engomi, Cyprus

The 5th International Multithematic Scientific Bio-Medical Congress 2017 was held at the European University Cyprus (EUC), Nicosia, Cyprus. EUC is becoming an Institution of excellence both in research and academia always aiming at new frontiers of innovative challenges. This annual congress that has now been globally recognized and subtitled ‘Bio-medical Scientific Cyprus (BSC)’ was founded and established by Professor Dr loannis Patrikios, a faculty member of the School of Medicine at EUC.

The key note speaker, the Distinguished Professor Dr Lee Hartwell (Nobel Laureate in Physiology or Medicine, 2001 for “Cell Cycle Control”) gave the presentation entitled “Educating physicians and scientists for the future”, via a live broadcast. He discussed the importance of being up-to-date with the growing body of research, in order to remain well-informed for the good of the future patients. It was emphasized to all students that regardless of their area of interest, pursuing the unknown is the key to discovery and achievement.

David Bates spoke about dietary factors in multiple sclerosis (MS). He pointed out various research reports suggesting that poly-unsaturated fatty acids (PUFA) are able to play a major role in the demyelination process of MS by reducing inflammation. He discussed the importance of dietary antioxidants as synergistic agents along with PUFAs and their potential to diminish disease symptoms by targeting specific patho-mechanisms (i.e. free-radical quenching, ability to affect the COX, and LOX pathways etc) supporting recovery in MS. Professor Bates concluded that even though dietary supplementation with PUFA is safe, the therapeutic abilities of PUFA have to be further proved by large well-designed phase III clinical studies.

The keynote speaker Panayiotis Soucacos lectured on “Microsurgery in the New Era of Managing Tissue”. He discussed the careful consideration that has to be given in reducing ischemia, vessel injury in replantation, as well as the multiple factors involved in such procedures, including the teams required, debridement, bone shortening, osteosynthesis, tendon repair, vessel repair, and nerve repair. Composite tissue Allotransplantation—transplantation of a vascularized limb or components for reconstruction of deficit following trauma or tumor resection—was presented as a current reality in surgical intervention. The new frontiers of hand and face remodeling using microsurgery techniques and human organ transplantation (immunology) were also presented in detail. Finally, he highlighted the emerging techniques and the massive needs nowadays for the aforementioned procedures.

Filippos Triposkiadis presented a talk entitled “Left Ventricular Ejection Fraction: An index of Left Ventricular Systolic Function” where he gave an overview of several factors leading to a decrease in left ventricular ejection fraction in heart failure. At first, he explained and discussed the Myofiber orientation in the left ventricle and how it changes smoothly from a left-handed helix in the subepicardium to a right-handed helix in the subendocardium and he continued by describing the overall wringing motion of the left ventricle during systole. Then he presented the recommendations from the European Society of Cardiology, the Guideline Treatment Algorithm, 2016, for Heart Failure.

Stavros Konstantinides continued the session lecturing on “Acute Pulmonary Embolism (PE)”. He specifically discussed that patients with intermediate-high risk of PE must be monitored in the ICU over the first 2–3 days. These patients may initially be treated with anticoagulation alone, but reperfusion treatment must be instituted as soon as hemodynamic or respiratory decompensation appears imminent. Syncope, tachycardia, and severe hypoxemia, are strong indicators of cardiorespiratory decompensation as he clearly stated.

Ingeborg Friehs presented data on “Mitochondrial Transplantation as a Novel Therapy for Specific Cardiac Diseases”. She spoke on how impaired mitochondrial function is associated with ischemia/reperfusion (I/R) injury, as seen with cardiac surgery and myocardial infarction and cardiac hypertrophy. She documented on her Preclinical studies of replacing mitochondria as well as on the ways of stimulating mitochondrial biosynthesis and the injection of cells to induce myocardial regeneration. As it was stated, rescuing the mitochondrial pool potentially prevents apoptotic cardiomyopathy death and delays the onset of cardiac failure.

Anastasis Stephanou continued the session on “Cancer and Immunotherapies”. He presented and discussed the effects of natural phytochemical extracts from Tripterygium wilfordii Hook F, graviola tree leafs, and amygdalin that have been found to be strong inducers of cell death in cancer cells, but not in normal cells. Moreover, in an attempt to identify possible targets, an in silico approach on the most abundant molecules from the above extracts indicated selective effects on Na + /K + ATPase and SERCA ATPase channel activity. As discussed, such data indicated that these natural phyto-compounds may have distinct target specificity expressed in tumors, but with limited toxic effects within the normal cells.

Barbara Seliger open the session on immunotherapy treatments and spoke about “Immune Escape of Tumors: Emerging Concepts and Therapeutic Opportunities”. She discussed the different modes of immunotherapy and stated that a reduced immune response may be due to immune escape and further understanding of the immune mechanism is needed to overcome immune evasion and tumor barriers. HLA class I alterations may be a frequent process and driving force for tumor evolution as she stated. She further discussed the oncogenic and IFN signaling as important for modulating HLA class I expression and the involvement of miRNA and RBPs in the process. Professor Seliger introduced MSA as a novel drug for reversion of MHC class I deficiencies.

George Chrousos gave a presentation entitled “Stress, Genetics and Epigenetics, and Human Evolution and Development.” He began with a description of Genetics vs Epigenetics, and complex systems being affected by disturbing forces, and continued on the evolutionary and developmental stressors such as starvation, injuries, adversaries, and social disruptions. He further highlighted the Epigenetic functions and how a malnourished mother (in third trimester vs 1st trimester) can lead to a reduced birthweight child with possible further implications on the said child’s future offspring.

Leondios G. Kostrikis spoke about HIV-1 disease from biology to chemotherapeutics and beyond. He stated that a human HIV-1 vaccine remains a central component in the quest to control the worldwide epidemic. Despite heroic efforts, the development of a safe and effectively preventive HIV-1 vaccine remains elusive. The design of immunogens able to elicit broadly neutralizing antibodies (bNAbs) is the holy grail of HIV-1 research. His research, as he discussed, deals with a novel vaccine strategy using inoviral-associated vectors to create stable “live” HIV-1 vaccines that can be sufficiently long-lived to elicit bNAbs at mucosal sites of HIV-1 transmission.

Marios Pantzaris in collaboration with Ioannis Patrikios gave a talk on Neuroaspis plp10, a cocktail of PUFA along with antioxidant vitamins. He explained how PUFA’s play a fundamental role in inflammation and immunity in relation to the peripheral and the central nervous system, in favor of modulating/reducing the inflammation process. He also discussed the metabolites of PUFA’s, the Lipoxins and Resolvins, that are as specifically involved in the resolution of inflammation. The role of nuclear respiratory factor and peroxisome proliferator-activated receptor in reducing inflammation was also discussed. This study indicated the potential implication of PUFAs in cardiovascular diseases, cancer, and neurodegenerative diseases, such as MS.

Nektarios Tavernarakis presented a talk entitled “Autophagic Pathways in Health and Disease: Mitophagy and Neurodegeneration”. He discussed how enhancing the accumulation of mitochondria shortens lifespan and conversely blocking accumulation of mitochondria promotes longevity. He presented data on mitophagy-deficient animals experiencing oxidative stress and he discussed that oxidative stress perturbs the distribution in mitochondria and in neuronal compartments. Moreover, he proved enhanced loss of dopaminergic neurons upon impairment of mitophagy and discussed findings of mitophagy impairment in Alzheimer’s disease models and in neurodegeneration.

Tassos Georgiou discussed Stargardt’s disease, presenting preclinical studies on ABCA4−/− mouse model as well as the clinical observational studies treated with Omega-3 fatty acids. He also presented the results of research team studies with regard to macular degeneration (with preclinical studies on CCL2−/− mouse model of macular degeneration). He concluded that supplementation with Omega-3 fatty acids suggests not only a protective mechanism in patients with Stargardt’s disease and macular degeneration, but also vision improvement.

In this meeting report, we have summarized the major scientific findings from the aforementioned presenters and their respective topics. We note that unpublished data are also included.

